# Humor appreciation can be predicted with machine learning techniques

**DOI:** 10.1038/s41598-023-45935-1

**Published:** 2023-11-03

**Authors:** Hannes Rosenbusch, Thomas Visser

**Affiliations:** 1 Department of Psychological Methods, University of Amsterdam, Amsterdam, The Netherlands; 2 Appinio GmbH, Hamburg, Germany

**Keywords:** Psychology, Scientific data, Statistics

## Abstract

Humor research is supposed to predict whether something is funny. According to its theories and observations, amusement should be predictable based on a wide variety of variables. We test the practical value of humor appreciation research in terms of prediction accuracy. We find that machine learning methods (boosted decision trees) can indeed predict humor appreciation with an accuracy close to its theoretical ceiling. However, individual demographic and psychological variables, while replicating previous statistical findings, offer only negligible gains in accuracy. Successful predictions require previous ratings by the same rater, *unless* highly specific interactions between rater and joke content can be assessed. We discuss implications for humor research, and offer advice for practitioners designing content recommendations engines or entertainment platforms, as well as other research fields aiming to review their practical usefulness.

## Introduction

What makes things funny? For millennia, philosophers and empirical scientists have pursued this question. Consequentially, we now have many theories, often backed up by experimental evidence, that highlight who labels what funny under which conditions. Despite these academic insights, the world outside the lab is certainly not freed from bad jokes, and likely never will be. What can humor research do to optimize humor exposure, for instance, on content platforms? To optimally guide practitioners, we set out to assess the usefulness of amusement predictors forwarded in academic research.

Specifically, we will quantify the empirical accuracy with which humor research can predict amusement by combining prominent psychological constructs with cutting-edge prediction models from the field of machine learning^1^. If decades of empirical research are enabling accurate predictions of funniness, research could focus on optimizing real-life applications. Conversely, if we are *not* able to predict humor appreciation accurately, we need to focus on constructing theories and predictors with higher predictive value. Therefore, the current work also ranks relevant constructs from most to least predictive.

In the current work, we focus on predicting perceived funniness, a component of humor appreciation. The term amusement is the emotional “state of experiencing something funny”^2,3^. Other components of humor appreciation like perceived offensiveness or boringness are not investigated here^4,5^.

## Predictors of amusement

Factors that influence whether someone will be amused by a humor stimulus can be divided into different categories. One of these categories is the nature of the joke, including its content and structural qualities^4,6^. Early theories suggested that jokes with certain contents are funnier than others because they tie in more closely with our supposed reasons for laughing. For instance, Freud suggested a cathartic function of jokes and laughter, as a valve for releasing tension from societally suppressed drives towards sex and aggression^7^. Thus, a hypothesis was formed (and sometimes supported^8^) that sexual or aggressive humor stimuli are funnier than others. In fact, even earlier theorizing suggests that all instances of humor consist of a satisfactory domination of others^9,10^. Other theories posit that people are more likely to laugh, when a joke elicits a strong but manageable cognitive violation (for a distinction with surprisingness/incongruity, see^11^) or when a joke has a strong elaboration potential (i.e., many funny implications^12^). To zoom out, such theories state that some jokes are simply funnier than others; they imply that to predict amusement, one should evaluate how *good* the respective joke is.

There is parallel stream of empirical evidence, sometimes coming from the same groups of researchers, showing that characteristics of the audience members are also predictive of humor appreciation. In short, some people will laugh at most jokes, whereas others remain hard to amuse^13^. One of the most differential traits when it comes to humor appreciation is a person’s dispositional cheerfulness, the temperamental basis for humor, often used synonymously with the fragmented term ‘sense of humor’^14^. Much like a person’s mood (e.g.,^15^), cheerfulness has both a dispositional baseline as well as time-varying components that have been observed to be aligned with people’s appreciation of humor stimuli^13,16^.

Other prominent personality constructs that have been linked to a person’s general level of humor appreciation include the Big Five personality traits. Specifically, extraverted^17–21^, conscientious^17,20^, open-minded^17,20^, and emotionally stable people^17,19,20,22^ have all shown heightened levels of humor appreciation, albeit not consistently. These positive relationships are likely restricted to personality-*coherent* humor stimuli (e.g., neurotic people specifically disliking absurd humor^6,17^). Similarly, sensation-seekers appreciate nonsensical humor, whereas their counterparts prefer humor with a clear uncertainty-resolving punchline^23,24^. The proposed explanation of the authors highlights that nonsense humor involves stronger cognitive stimulation, thereby dividing sensation-seekers and avoiders. Comparable to humor appreciation, humor production is divided across personality dimensions. For instance, extraverts produce more affiliative humor, whereas agreeable people tend to produce non-aggressive humor^25,26^.

Next to mood and personality, people’s capabilities can predict humor appreciation. Specifically, people with a higher competence for producing funny jokes^20^ rated humorous cartoons comparatively low, potentially due to a heightened desire for dominance (humor production) as opposed to submission/affiliation (humor appreciation). Thus, a broad string of research focuses on the attributes of the audience when predicting amusement, rather than merely scoring the content of the joke.

Note that some of the listed findings go beyond simple person-effects and advance to the interaction between person characteristics and the characteristics of the joke (e.g., an aggressive person enjoying an aggressive joke). Similarly, work focusing on stimuli differences often acknowledges the notion of interactivity. For instance, Freud himself gave examples of people differing in their habitual suppression of urges which in turn affected their sense of humor^7^. Proposed advantages of specific joke contents, like sex and violence, were merely due to the observation that the suppression of these urges were common, thus rendering such joke contents more effective on average. In short, no theory on humor appreciation claims that it is exclusively the joke, or exclusively the nature of the listener that determines amusement. The question is merely, to which degree the two sources and their interaction contribute.

In a variance-decomposition study, ICC values ascribed higher importance to raters than to stimuli when it came to funniness ratings, and even more variance by pooling variance for specific rater X stimulus pairs^27^. This suggested that the totality of interaction effects harbors the highest predictive power, but it remains to be determined which specific effects account for this finding, and how accurate resulting prediction models are, especially when applied outside the training sample. The importance of person-stimulus interactions is also highlighted in some humor appreciation theories like the Benign Violation Theory which posits that amusement requires a simultaneous categorization of jokes as violating and benign^11,28^, both judgments being influenced by the rater’s specific relationship to the joke content^29^. In the current set of studies, we review the practical usefulness of person-level predictors, stimulus-level predictors, and their interactions, when building cutting-edge machine learning systems trained to predict people’s appreciation of humor content.

## Predictive accuracy as practical relevance

Traditionally, psychological sciences focus on offering coherent explanations for human behavior. Usually, this is achieved by accumulating and mapping empirical observations to causal theories. Many such theories imply patterns of statistical associations between two or more relevant measurements. While theories often state that one measure should ‘predict’ the other (i.e., a non-zero statistical association), there is usually no pre-defined cut-off for how *accurate* this prediction should be to support the theory.

For example, various findings suggest that bad quality of sleep is associated with depression and suicide^30^. A theorist might wonder about the mechanism and directionality of the association. Conversely, a practitioner might wonder whether it is now feasible to build the researchers’ statistical model into their fitness tracker app to enable an “early-warning” feature for poor sleepers^31^. If the model can accurately predict depression risk for new participants, one could even consider a direct alert going out to local health care professionals, similar to car crash detection in mobile phones. However, most analyses in psychological research do not inform practitioners about the predictive accuracy that their theory affords^32^. Integrating machine learning models and, more importantly, out-of-sample accuracy assessments would therefore constitute a clear step from theoretical to practical usefulness^33^.

Regarding humor, most machine learning systems are focused on humor *detection*, meaning that models automatically infer which texts or images were meant to convey humor^34–37^. Interestingly, state-of-the-art performance in humor detection is achieved by models that are designed based on psychological humor theories^38^. While these research efforts do not focus on humor *appreciation*, they highlight that computational and psychological research can mutually reinforce each other when analyzing humor stimuli.

Some research also exists on machine predictions of humor appreciation. Specifically, models are trained to predict which of two humor stimuli will be rated as funnier, when aggregating ratings of multiple participants^39^. The efforts are often only partially successful meaning models make many mistakes in ranking the funniness of humor stimuli^40,41^.

In this work, we change the level of analysis to pairings of individual raters with individual stimuli (i.e., no aggregation). Ruch reviewed the stark effect of the aggregation choice on later results (e.g., meaning and magnitude of correlations between funniness ratings and auxiliary variables^42^). Generally, aggregation boosts reliability and thus our focus on single experiences of humor by single individuals makes prediction more difficult. However, a compensatory advantage is that individual-level characteristics of both, the stimulus and the rater, can be used for the prediction of amusement.

The benefit of reviewing the predictive power of humor research is that practitioners can use the new insights to build content recommendation websites, revise their comedy routine, or forecast the success rate of their clinical humor treatments. Generally, it gives insight into the practical usefulness of the many variables that appear in recent humor theories.

## Study 1

We assess whether prominent constructs from humor research can accurately predict humor appreciation and which specific constructs offer the largest gains in empirical accuracy. All participants provided informed consent. The study procedure in accordance with the APA ethics code as well as the Declaration of Helsinki, and was approved by the Faculty Ethics Review Board of the first author’s research institution under leadership of Lourens Waldorp.

### Method

#### Procedure

In an initial survey, participants filled out a questionnaire assessing constructs relating to personality, morality, ability, humor attitudes, and demographic data. Two days later, they received an invitation to a second survey which assessed their current mood as well as humor appreciation for 10 stimuli. It was further asked how well participants understood the English texts of the humor stimuli. All participants passed an attention check asking for the sum of eight and two. The data from the initial study was merged with the second survey using the participants’ unique user ID on the platform.

#### Materials

When trying to estimate the predictive value of psychological research it is of central importance to select promising predictor variables. All constructs and variables outlined below were selected based on their prominence in the literature (cf., sources above, and per measure below). Some noteworthy omissions are discussed in the limitation section. We relied on published short versions of psychological scales. All stimuli and newly generated questions about people’s attitude towards the content of the humor stimuli are included in the Supplementary materials.

##### Big Five

Various meta-analyses and reviews of the relationship between the Big Five personality traits and humor have been conducted^25,26^. The Big Five Inventory 10 (BFI-10) is a ten-item version of The Big Five Inventory (BFI) developed by Rammstedt and John^43^ and measures agreeableness, extraversion, openness to experience, conscientiousness, and neuroticism. Answers are given on a 5-point scale (“Disagree strongly” to “Agree strongly”). The scale authors report an average six-to-eight-week retest-reliability of 0.75.

##### Cheerfulness

In humor appreciation, state cheerfulness is one of the central constructs as it forms part of the temperamental basis for humor^44^. The State-Trait Cheerfulness Inventory State Version–Short Form (STCI-S18^45^) is a short version of the State-Trait Cheerfulness Inventory State Version (STCI^16^). It is an 18-item survey that measures cheerfulness on three dimensions: cheerfulness, seriousness, and bad mood. Because mood is already measured separately, only the 6 cheerfulness items were used here. The answers to these items were given on a 4-point scale (“Strongly disagree” to “Strongly agree”). The authors report a four-to-five-week retest-reliability of 0.85. The Cronbach's alpha coefficient in the current study was 0.77.

##### Moral identity

In humor research, a negative effect of morality on humor appreciation was observed by various groups^46–48^ for at least some forms of humor. The five-item internalization dimension of the Moral Identity Scale^49^ was developed to measure moral beliefs. The participant is asked to imagine a person that is “caring, compassionate, fair, friendly, generous, helpful, hardworking, honest, kind”. After this, five identification items are answered on a 5-point scale (“Strongly disagree” to “Strongly agree”). The original authors report a retest-reliability of 0.49 over a four-to-six-week time period, arguing that the construct varies over time. The Cronbach's alpha coefficient in the current study was 0.7.

##### Sensation seeking

While the relationship between dispositional sensation-seeking and humor appreciation is sometimes positive and sometimes negative (depending on the nature of the humor stimuli) it remains a potent predictor of humor appreciation across cultures^23^. The Brief Sensation Seeking Scale (BSSS^50^) is an eight-item survey created to measure the components of sensation seeking. The answers to the BSSS are given on a 5-point scale (“Strongly disagree” to “Strongly agree”). The original authors of the scale did not report a retest-reliability but a Dutch version achieved a two-week retest reliability of 0.93^51^. The Cronbach's alpha coefficient in the current study was 0.81.

##### Humor production

Moran and colleagues^20^ found that people that were better at producing humor, were less appreciative of humor stimuli than others (see^19^ for a set of opposing results). The Multidimensional sense of humor scale (MSHS^52^) assesses respondents’ self-rated skill in being funny. The relevant subscale encompasses four items. Answers are given on a 5-point scale (“Strongly disagree” to “Strongly agree”). The original authors did not report a retest-reliability, but a Finnish version showed a three-year retest reliability for the subscale of 0.75^53^. The Cronbach's alpha coefficient in the current study was 0.75.

##### Mood

It is an ongoing question which mood facets influence humor appreciation (the most^13,15,54^). The International Positive and Negative Affect Schedule Short Form (I-PANAS-SF^55^) is a short version of the Positive and Negative Affect Schedule (PANAS^56^). It is a 10-item measurement in which people rate how they generally feel (e.g., “upset”, “nervous”) on a 5-point scale (“never” to “always”). The authors report an eight-week retest-reliability of 0.85.

##### Stimulus-specific attitudes

Broad traits, as listed above, are prominent in humor research, but narrow constructs can be highly predictive as well if they fit the context well. In the case of humor appreciation this pertains specifically to people’s attitudes towards specific joke contents^57^. Knowing whether someone is anti-Trump will likely be informative for their amusement from anti-Trump jokes, albeit less informative for broader humor categories. Here, we assess stimulus-specific attitudes with newly developed questions about the specific humor stimuli displayed in the survey Examples of these are “I like pictures of cute animals” or “I am very familiar with Game of Thrones”. The respective jokes included a humorous picture of a dog and a meme featuring a scene from Game of Thrones. Attitude items were rated on a 5-point scale from (“Strongly disagree” to “Strongly agree”).

##### Humor appreciation

The prediction target, humor appreciation, was measured for ten different humor stimuli with two items per stimulus. These stimuli were selected from a previously published corpus of 105 diverse humor stimuli^27^. The selected humor stimuli were categorized by the original authors as falling into five categories (there were more in the corpus): affiliative, self-defeating, aggressive, self-enhancing, and sexual^58,59^. For the current study, one textual and one image stimulus were selected from each category.

Note that the assignment of the stimuli into one of the five categories is not objective. The original authors write “Note that most stimuli fell into multiple categories […] because everyday humor usually combines different dimensions” (^27^, p. 1390). Table 1 shows an example stimulus for each of the five categories. Note, for instance, that jokes which weren’t assigned to the category ‘aggressive’ still vary in their aggressiveness.

**Table 1:** Example stimuli.

Category	Stimulus
Affiliative	"Knock, knock. Who’s there? Olive. Olive, who? Olive you, and I don’t care who knows it"
Self-defeating	"It’s true that I’m CUTE: C(ringy), U(nattractive), T(rash), and E(asy to forget)"
Aggressive	"I'm not saying I hate you, but I would unplug your life support to charge my phone"
Self-enhancing	"Can a kangaroo jump higher than the Empire State Building? Of course. The Empire State Building can't jump"
Sexual	"What’s the difference between a G-spot and a golf ball? A man will actually search for a golf ball"

Text formatting was simplified for the table.

Here, humor appreciation was measured with two questions: “How funny do you find this {text/image}?” (1 = “not funny at all” to 7 = “very funny”^27,48,57^, and “Final evaluation:” with answer options “Good joke” and “Bad joke”). We selected a continuous and a binary measure to have complementary accuracy metrics available during the out-of-sample accuracy analysis.

#### Sample

A German panel provider offered participation through their mobile application where anonymous users can generate and distribute their own opinion polls, as well as answer polls of others. The app includes gamified rewards such as levels and coins that can be gathered by generating and participating in surveys. Once a certain number of coins is reached, participants can donate 10 Euro to protect the rainforest or get 10 Euro transferred to their PayPal account. However, the main driver for participation is entertainment rather than monetary rewards, as well as the opportunity for anyone to conduct representative polls outside one’s own social bubble^60^. Participants from Germany (58.87%), the UK (24.79%), and the USA (16.33%) entered the study for a total of 5473 responders (50.23% male, 49.77% female). Ages ranged from 18 to 84 (M_age_ = 32.68, SD_age_ = 10.76). All participants indicated in a previous assessment that they can read and speak English and passed an English attention check question.

#### Analysis plan

The ultimate goal of the current work is to assess *how accurately* one can predict humor appreciation on unseen cases, and *which predictors* are the most useful in that regard. Thus, the focal pieces of the current study are prediction models relating humor appreciation to a range of predictors from psychological research. Given the tabular nature of the data, we utilized boosted decision trees (XGboost^61^) as the prediction model (ensemble). The XGboost algorithm is currently considered the state-of-the-art technique for predicting target variables based on tabular data^62^. Tabular data refers to the data commonly found in social science investigations with multiple observations scored on qualitatively distinct variables which can be organized in a table-like format. Other collections of data include images, audio, or text, for which other approaches, most notably neural networks, trump the performance of tree-based algorithms like XGboost (for a review of both approaches see^63^).

Much like in OLS regression models, the XGboost algorithm relates predictor variables to an outcome variable, and its inner parameters are optimized for prediction accuracy. However, rather than fitting a linear combination of predictors, XGboost is based on decision trees that funnel observations down different “branches” based on logical statements (e.g., “Age > 30” goes left, other datapoints go right). These splits are optimized to create end nodes (i.e., “leaves”) on which data points have similar scores on the outcome variable. The XGboost algorithm then iteratively introduces new trees which predict the residuals of previous trees rather than the raw target scores. Thereby, the predictions of the summed tree outputs approximate the target score while actively combating mispredictions. The added complexity of the algorithm usually results in substantial improvements of prediction accuracy compared to OLS regression. For details and implementation guidance, see González and colleagues^64^.

The entire pipeline of data transformation, model tuning, and final analysis was conducted separately for the continuous measure of humor appreciation (metrics: *R*^*2*^*,* RMSE) and the binary measure (metrics: AUC, accuracy). Data was transformed into a long format with a single response per row (54,730 rows). One response per participant was randomly selected to form part of the test set which was only accessed once during final evaluation (5473 rows). We used tidymodels and its associated packages^65^ in R^66^ for both tuning and evaluating the models. We used tenfold cross-validation to assess the performance of 42 hyperparameter settings and selected the best performing setting to fit the model on the full training data. We compute variable importance and final performance on the test set. Variable importance scores were generated by permutating the values for individual predictor variables and quantifying associated model performance drops in the test set. The generation of all results was done in the same way but separately for three sets of predictors: (1) all psychological constructs (including demographic info), (2) average ratings of respectively the current joke and of the current participant, and (3) the combination of both of these predictor sets. Responses in the test set were excluded when computing rating aggregates. All data and scripts are in the Supplementary materials. We compare model accuracies by inspecting 95% bootstrap confidence intervals. Formal tests for comparing the predictor sets were not necessary given the width and separation of intervals (see below).

### Results

**Figure 1:**
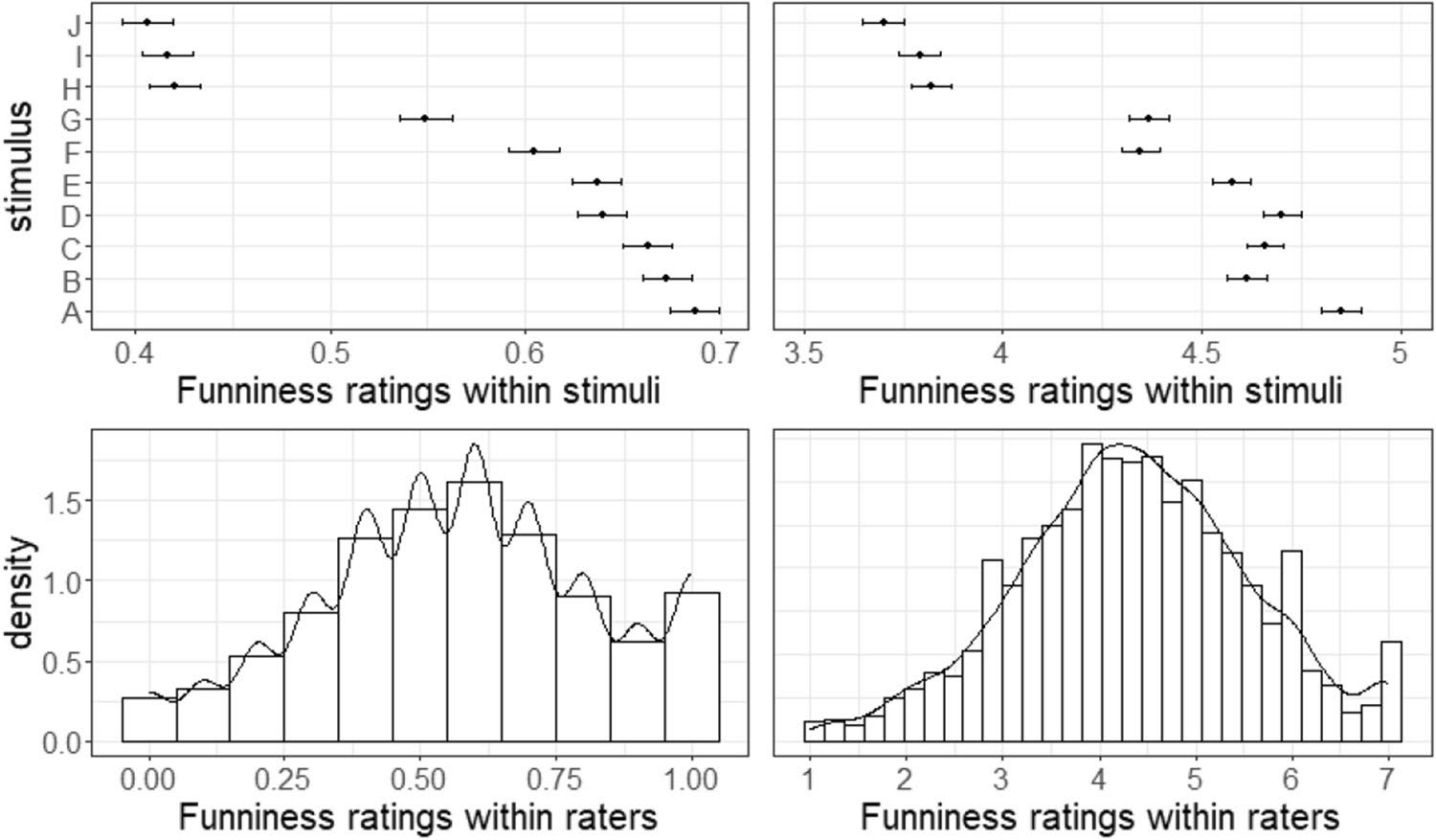
Top left: Proportion of ‘Good joke’ ratings for each stimulus including 95% confidence intervals (binary scale). Top right: Average funniness ratings for each stimulus including 95% confidence intervals (7-point scale). Bottom left: Each participant made ten binary ‘good vs bad joke’ decisions averaged into a personal proportion score (0–1). The histogram shows these proportion scores for all participants. Bottom right: Each participant provided ten continuous funniness rating averaged into a personal rating score (1–7). The histogram shows these average rating scores for all participants.

Distributions of humor appreciation for the binary measure (overall: 56.96% “good joke”) and the continuous measure (overall: *M* = 4.34, *SD* = 1.92) for each of the 10 stimuli are depicted in Fig. 1.

Hyperparameter settings for the XGboost algorithms were selected based on cross-validation performance and can be reproduced with the supplementary scripts. Table 2 shows all accuracy metrics of each model when predicting humor appreciation.

**Table 2:** Accuracy achieved with different predictor sets.

Predictors	Accuracy	AUC	RMSE	R^2^
Scales	0.642	0.640	1.648	0.274
Rating aggregates	0.687	0.677	1.590	0.325
Scales + rating aggregates	0.705	0.695	1.554	0.361

*AUC* Area under the receiver operating characteristic curve; 95% confidence intervals only include shifts by maximum 0.01 for all metrics.

The Supplementary material includes scripts and results for an alternative predictor setup, where individual survey *items* are used as predictors instead of the underlying psychological *scales*. The performance is virtually the same as shown here.

**Figure 2:**
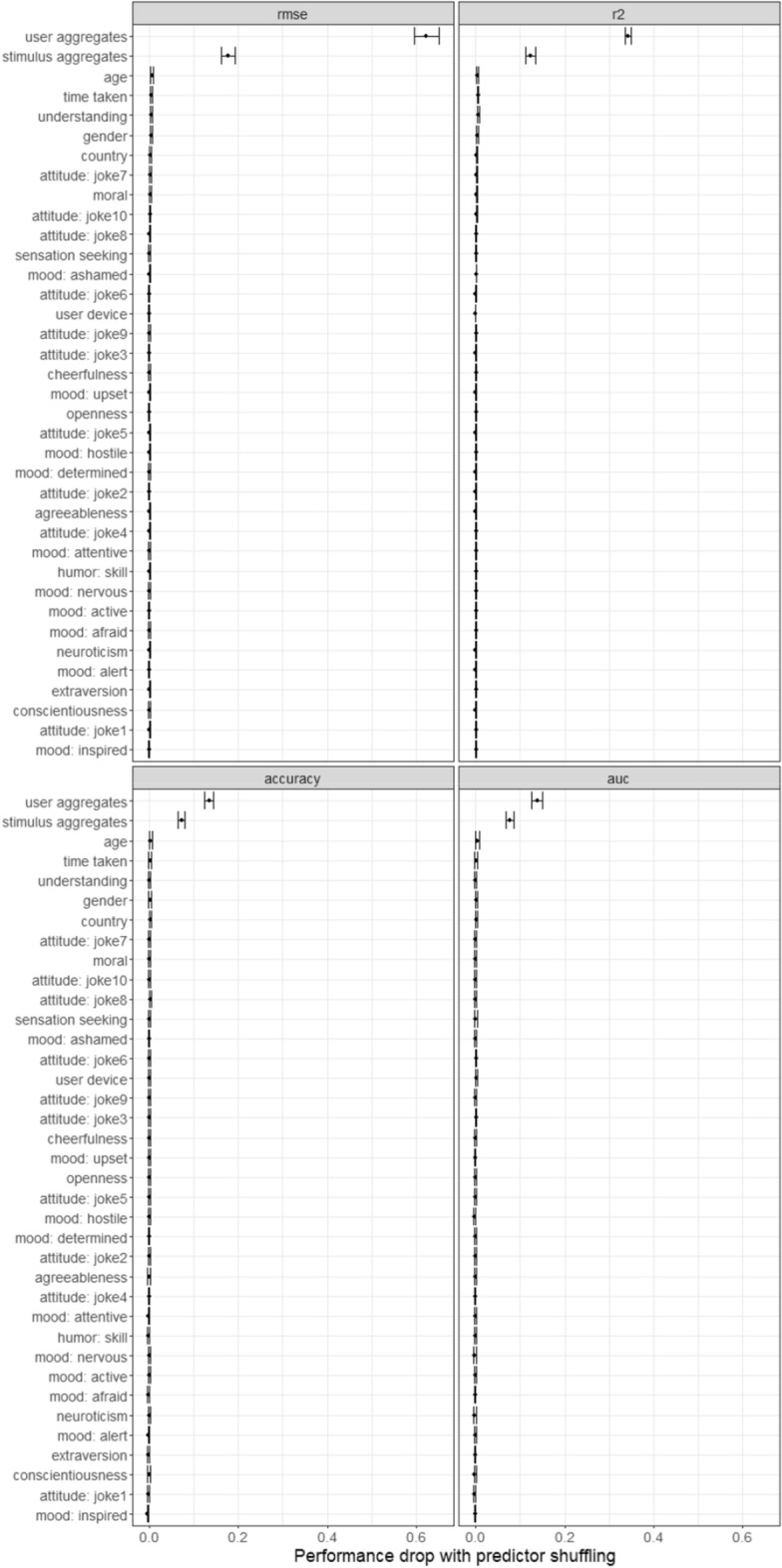
Drops in prediction performance when a given predictor variable is scrambled.

Variable importance scores (see Fig. 2) were quantified as average performance drops on the test set when randomly permutating predictor variables (100 iterations).

It is apparent that the model rests primarily on how other people rated the current joke, and the current rater’s appreciation of other jokes. Scrambling any of the other psychological constructs and variables leads to negligible deviations in model performance. If one assumes that participants responded to all the jokes equally, were not indicating their authentic appreciation of the stimuli, their responses could artificially inflate the predictive power of past ratings. However, after removing this group of 249 people (4.5%), and retraining the models, predictions were still primarily based on past responses.

**Figure 3:**
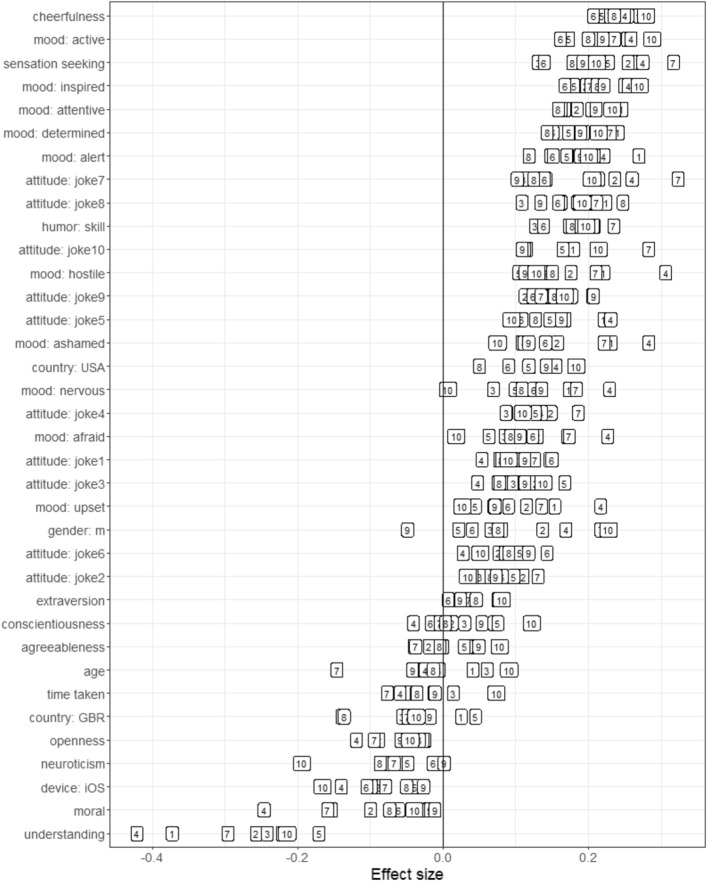
Statistical associations (Pearson correlations and Cohen’s D’s) between predictors and continuous humor appreciation scores. The content of the individual humor stimuli (boxes 1–10) can be reviewed in the Supplementary materials.

Figure 3 shows the zero-order associations between individual psychological variables from psychological research and the separate funniness rating for all 10 stimuli.

### Discussion

The accuracy with which humor appreciation could be forecasted was around 70% and *R*^*2*^ = 0.36. While this is substantially better than chance, predictions for individual people are thus far from reliable even when considering a wide variety of predictors from psychological research. While we calculated lower accuracy limits by establishing guessing baselines, we could not quantify a higher limit that accounts for random (i.e., non-predictable) noise in the humor appreciation scores. Study 2 quantifies this limit as the retest reliability of the predicted variable. This will clarify whether the remaining errors are due to weak predictors or unreliable measurements of humor appreciation.

A positive take-away for practitioners is that simple aggregates of a user’s past behavior on the platform (i.e., here operationalized as funniness ratings) were almost sufficient to achieve the maximal model performance with only small incremental gains coming from the assessment of psychological constructs. Before concluding that much psychological work has little incremental value for the prediction of humor appreciation there is an important caveat to consider; many psychological constructs were previously highlighted to matter because they *interact* with the nature of the material when steering humor appreciation. While the nomenclature differs, various research groups have found person-specific differentiation of stimuli into aggressive vs affiliative^58^, violating vs non-violating^28^, or adaptive vs maladaptive humor^67^. The resulting humor appreciation depends on a person’s unique appraisal of these attributes. In Study 1, we did not actively encode such rater X stimulus interaction terms warranting an extended analysis for predictor importance in Study 2.

Bi-variate analyses in Study 1 replicated much previous work. For instance, cheerfulness and mood were consistent predictors of higher humor appreciation^68^. Further, we also observe lower appreciation scores for people scoring high on morality^48^. A more surprising observation was that people finding the English language in the materials generally harder to understand, rated jokes funnier. This is counterintuitive as understanding (or “getting”) the joke is usually a strong predictor for finding it funny^69^. We assume that the current combination of sample (mostly native/fluent English speakers) and materials (no challenging vocabulary) led to virtually everyone understanding the jokes without any problems (median = 4; 5-point scale). Thus, the variation in self-indicated understanding could be mostly due to other factors like self-ascribed intellect. This surprising finding is followed up on in Study 2.

## Study 2: Pre-registered follow-up

In Study 2, we aim to address three questions remaining after Study 1:

- Can we predict humor appreciation more accurately by including rater X stimulus interactions?
- What is the reliability of the humor appreciation scores (i.e., the maximally achievable prediction accuracy)?
- Do we replicate the findings of Study 1 with new stimuli (including the finding that lower understanding of the stimuli is associated with higher stimulus appreciation)?

To that end, we conduct a pre-registered replication of Study 1, including different participants and humor stimuli. Further, we extend the measures to include interactions between rater and stimulus characteristics, Lastly, we estimate the reliability of humor appreciation scores through a test–retest procedure.

### Method

The procedure, assessed constructs (Cronbach’s alpha deviations from first study < 0.02), and analysis plan stayed the same as in Study 1 with four exceptions:

1. We used 10 new humor stimuli from the corpus of jokes from Rosenbusch and colleagues^27^. All stimuli are available in the Supplementary materials. Again, we picked one textual and one image joke from each of the pre-annotated categories of self-defeating, affiliative, aggressive, self-enhancing and sexual humor to ensure stimulus diversity^58,59^. Table 3 shows the text stimuli.
2. The general item “How hard was it for you to understand the English language/vocabulary in the jokes?” was replaced with 10 stimulus-specific items: “I understood this joke” (1 = strongly disagree, 5 = strongly agree). This was implemented to shed light on the observation from Study 1 that generally poorer language understanding was associated with more humor appreciation overall.
3. Two Likert-items per humor stimulus were added for each stimulus (“The joke seems aggressive” and “The joke seems friendly”; 1 = strongly disagree, 5 = strongly agree). These will be used to quantify the interaction between the nature of specific stimuli and the unique perception of each rater.
4. In a second wave, participants were invited to re-evaluate the same humor stimuli to allow for an estimation of re-test reliability of the humor appreciation measures. Notice, that we quantify the reliability of individual responses, as this aggregation-free reliability score poses the theoretical ceiling for the prediction models.

**Table 3:** Example stimuli.

Category	Stimulus
Affiliative	"Why did the bee marry? He’s finally found his honey"
Self-defeating	"No more self-deprecating jokes, I whisper fatly"
Aggressive	"Light travels faster than sound. This is why some people appear bright until you hear them speak"
Self-enhancing	"My calculator stopped working mid way through my exam. I can’t count on it anymore"
Sexual	"Do you need a stud in your life? Cause I got the STD and all I need is U"

Text formatting was simplified for the table.

#### Sample

Participants were collected through the same online survey platform as in study 1. Participants from Germany (67.48%), the UK (24.54%), and the USA (8%) entered the study for a total of 5502 responders (59.47% female, 40.53% male). Ages ranged from 16 to 99 (M_age_ = 29.58, SD_age_ = 10.06). All participants indicated in a previous assessment that they can read and speak English and passed an English attention check question. None of the participants from study 1 were admitted to study 2. In a smaller, second wave, the stimuli ratings were re-provided by 407 participants (52% female, 48% male). Ages ranged from 16 to 75 (M_age_ = 35.17, SD_age_ = 11.86). Of the re-invited participants, 90% provided their answer between four and seven days after the first wave.

### Results

**Figure 4:**
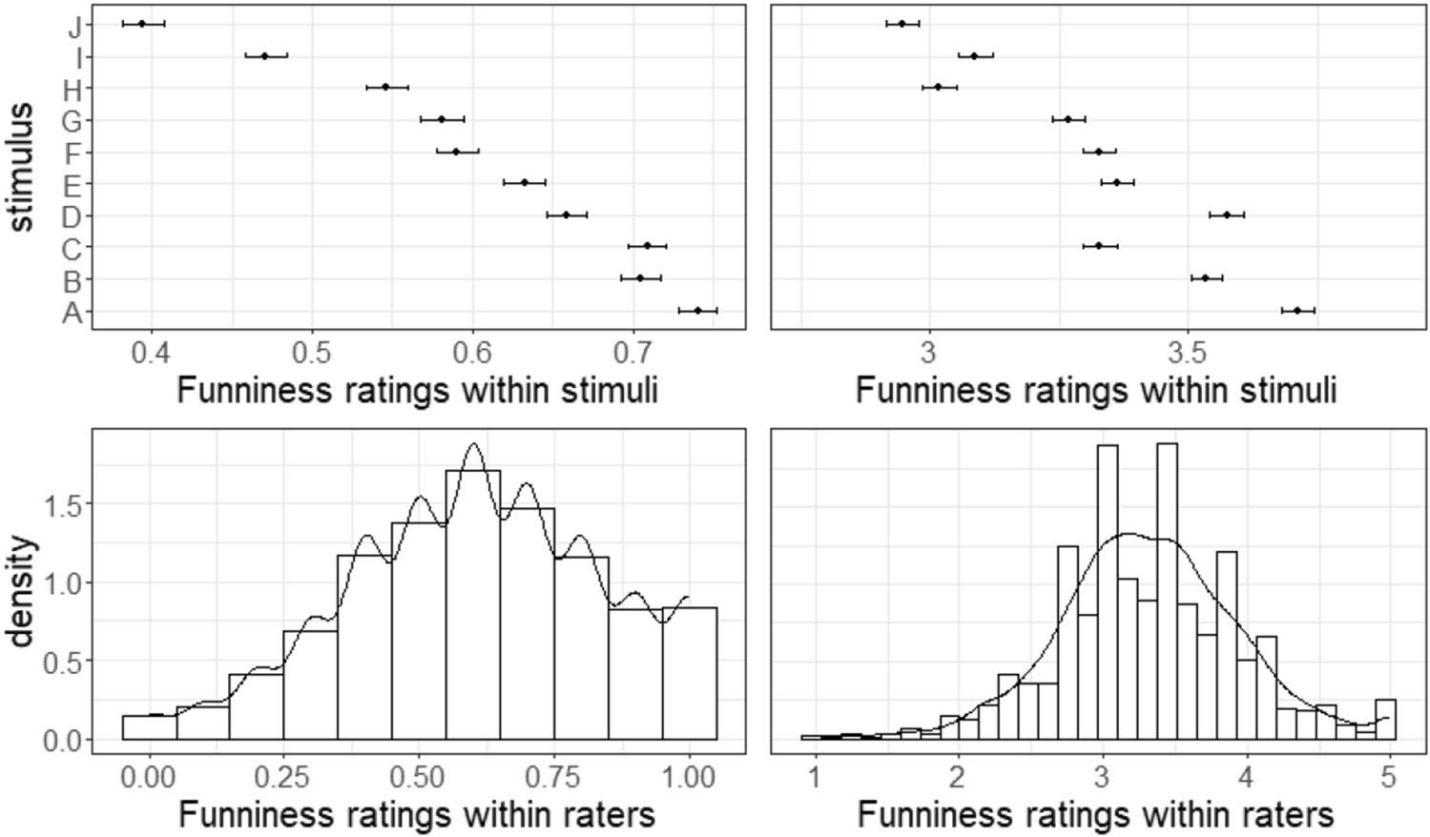
Top left: Proportion of ‘Good joke’ ratings for each stimulus including 95% confidence intervals (binary scale). Top right: Average funniness ratings for each stimulus including 95% confidence intervals (7-point scale). Bottom left: Each participant made ten binary ‘good vs bad joke’ decisions averaged into a personal proportion score (0–1). The histogram shows these proportion scores for all participants. Bottom right: Each participant provided ten continuous funniness rating averaged into a personal rating score (1–7). The histogram shows these average rating scores for all participants.

Distributions of binary humor appreciation scores (overall: 60.3% “good joke”) and continuous scores (overall: *M* = 3.32, *SD* = 1.21) for each of the 10 stimuli are depicted in Fig. 4.

**Figure 5:**
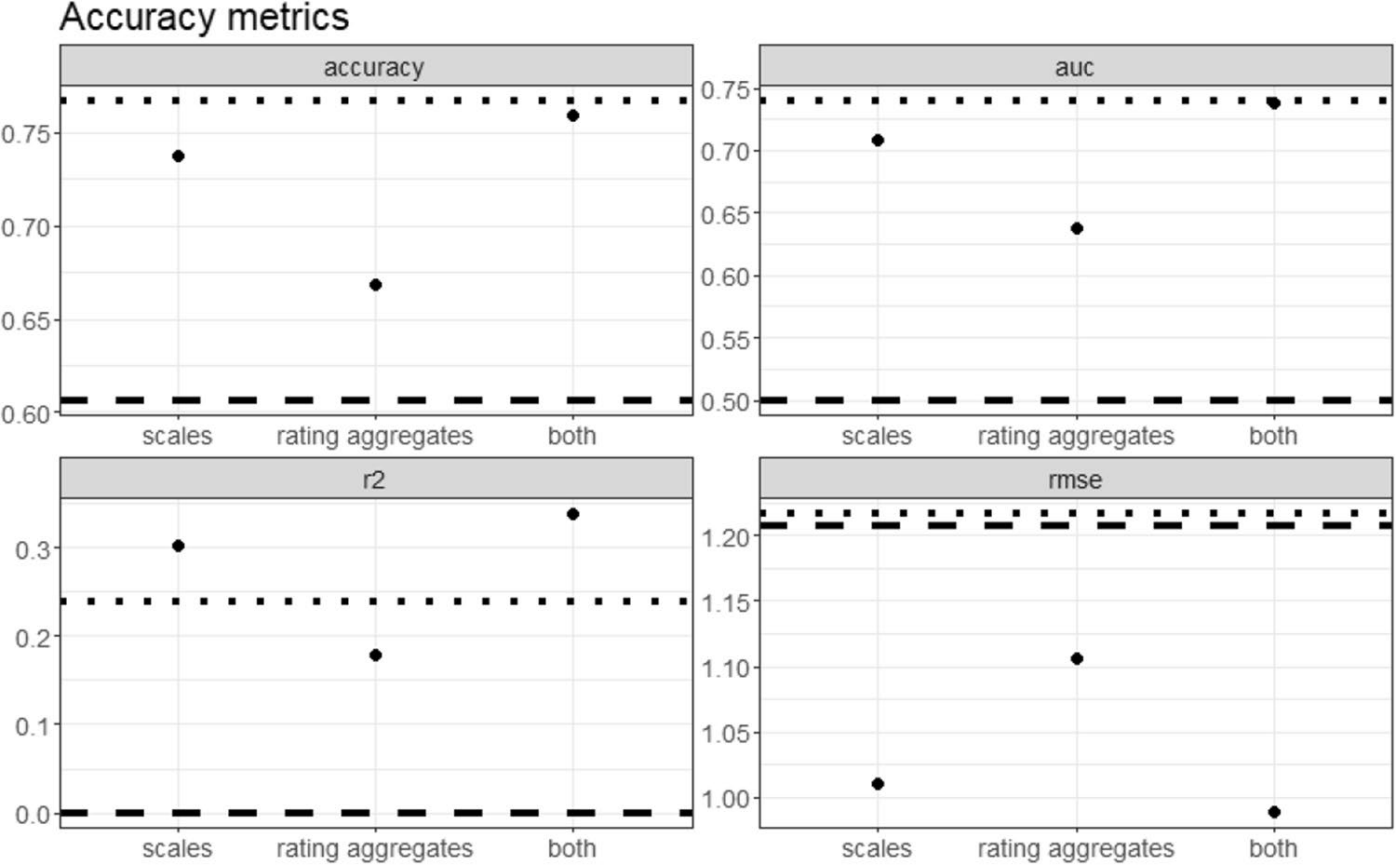
Out-of-sample prediction accuracies achieved with the three different predictor sets. The dashed line highlights for each accuracy metric which score would be achieved by simply always guessing the mean/majority class. The width of the points is wider than their respective 95% confidence intervals. The dotted line represents the retest-reliability of the outcome variable.

Hyperparameter settings for the XGboost algorithms were selected based on cross-validation performance and can be reproduced with the supplementary scripts. Table 4 and Fig. 5 show all accuracy metrics of each model when predicting humor appreciation on new data.

**Table 4:** Accuracy achieved with different predictor sets.

Predictors	Accuracy	AUC	RMSE	R^2^
Scales	0.737	0.708	1.01	0.303
Rating aggregates	0.668	0.638	1.107	0.178
Scales + rating aggregates	0.760	0.738	0.989	0.338

*AUC* Area under the receiver operating characteristic curve; 95% confidence intervals only include shifts by maximum 0.01 for all metrics.

When taking the retest-reliability of the humor appreciation as the theoretical ceiling of prediction accuracy, one can see that the prediction of binary appreciation scores (top panels) virtually reaches the maximal limit. For continuous appreciation scores, the machine prediction even surpasses this threshold. This is likely related to the low stability of continuous appreciation scores over time (*r* = 0.49). Humor appreciation varies from situation to situation, making a retest-reliability score a very strict measure of error-freeness. The issue of construct stability vs. measurement error is discussed below.

**Figure 6:**
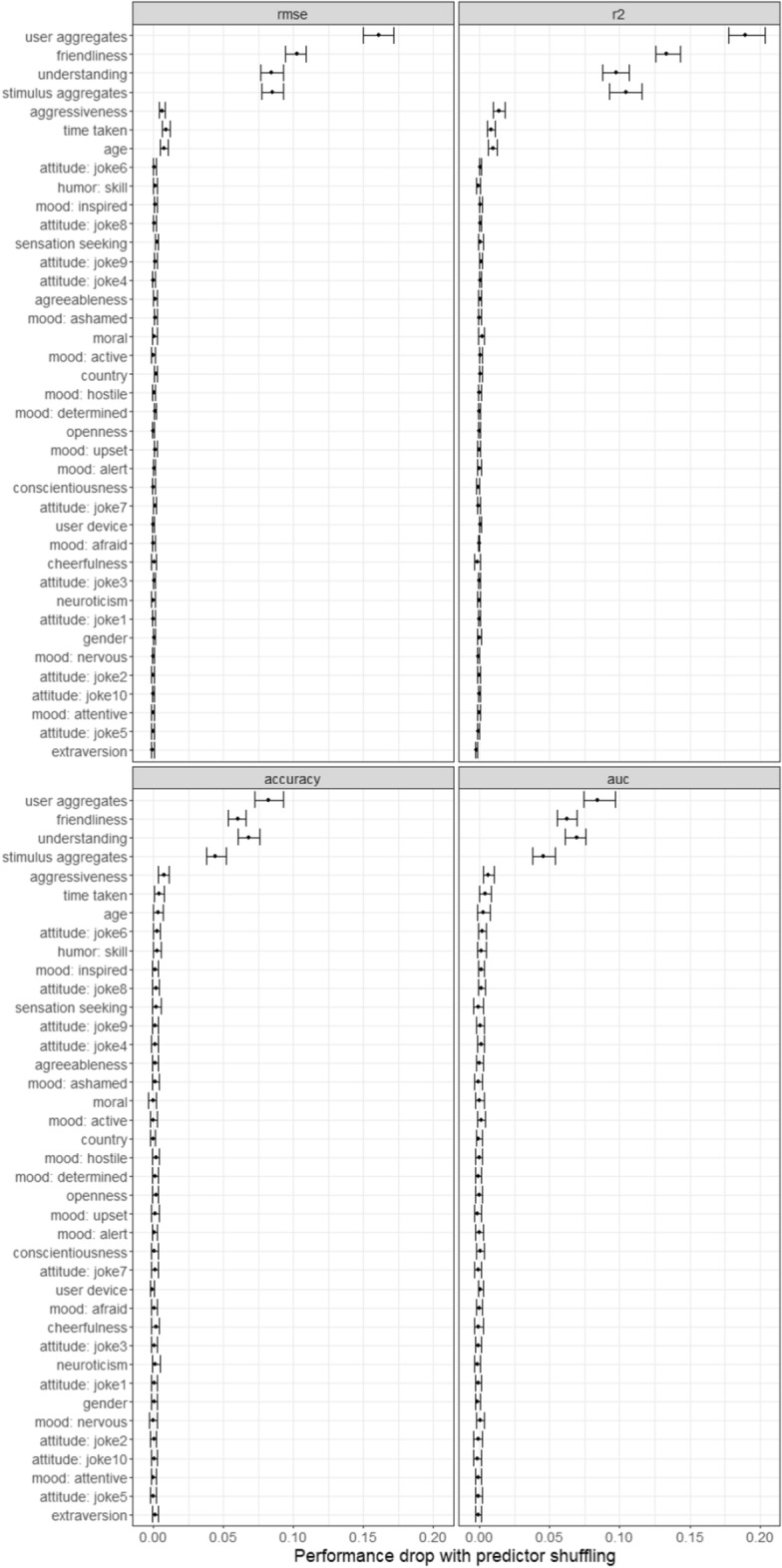
Drops in prediction performance when a given predictor variable is scrambled.

Variable importance scores (see Fig. 6) were quantified as average performance drops on the test set when randomly permutating predictor variables (100 iterations).

It is apparent that past appreciation by the participant (of other stimuli) and other participants’ ratings of the current stimulus remain potent predictors. However, the predictors of perceived friendliness, understanding, and aggressiveness—all rater X stimuli measures—score high as well. This explains why the accuracy of the scale-based model (including these variables) now surpasses the performance of the purely “past ratings”-based model.

**Figure 7:**
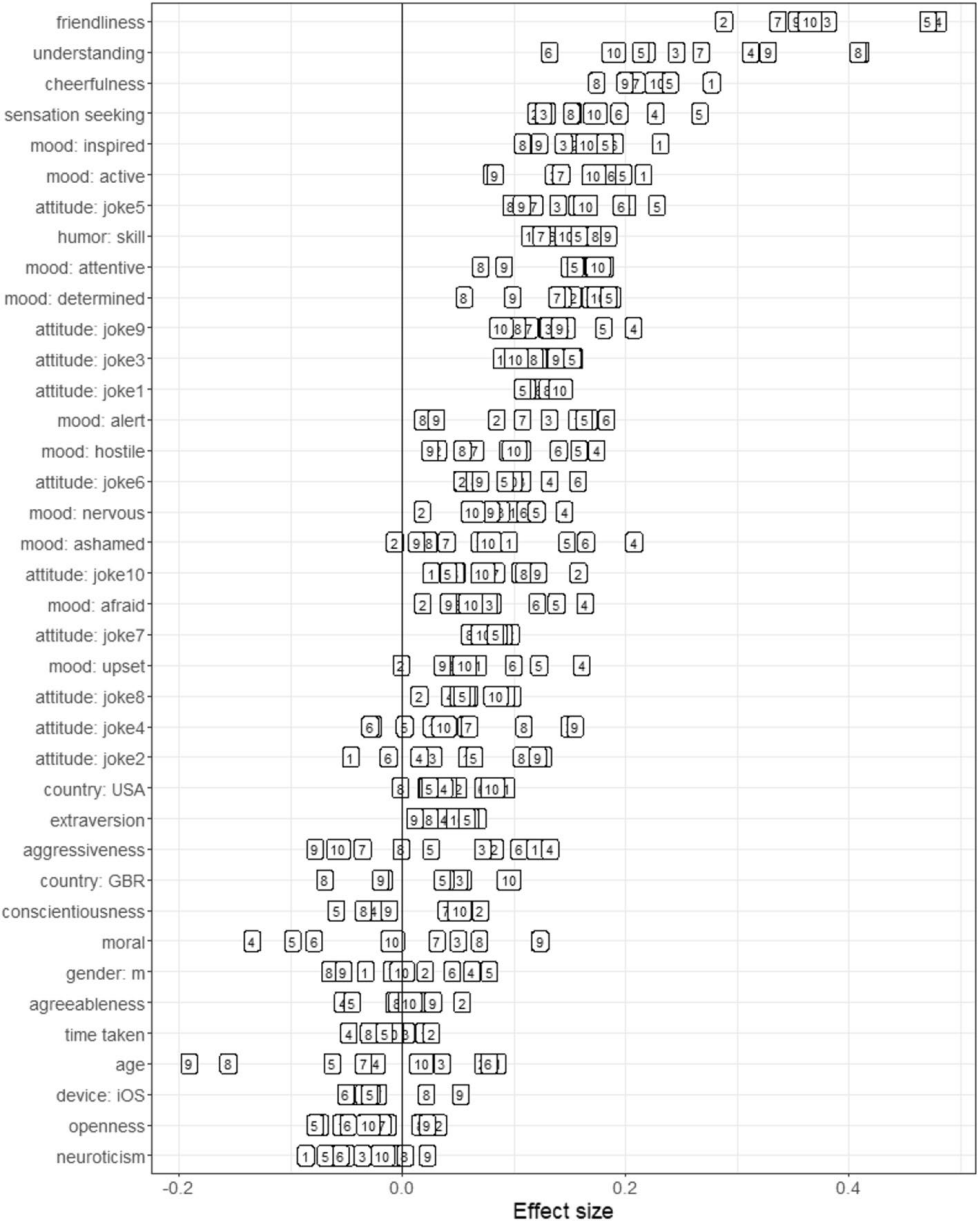
Statistical associations (Pearson correlations and Cohen’s D’s) between predictors and continuous humor appreciation scores. The content of the individual stimuli (boxes 1–10) can be reviewed in the Supplementary materials.

Figure 7 shows the zero-order associations between individual variables from psychological research and the separate funniness rating for all 10 stimuli.

In Study 2, where understanding of jokes was measured separately for each stimulus, the association between understanding and appreciating a joke is positive. This addresses the previously paradoxical finding that more (general) understanding predicts less (general) appreciation (cf., Study 1).

## General discussion

Imagine you just thought of a fantastic joke. There are 100 contacts in your phone, but you are usure who to tell the joke to. Unbeknownst to you, 60 of them would laugh and 40 would not. Thus, if you told the joke to a randomly selected person, you would have a 60:40 chance of success. Applying much of the psychological research on humor appreciation from the last decades would boost this chance to about 76:24, a noticeable improvement but far from certainty. An obvious catch is that one needs to closely analyze specific characteristics of your contacts to achieve this improvement. Specifically, our results highlight the predictive value of past amusement proclivity, above all other constructs. The strongest “laugher” among your contacts is likely a safe bet. The next best class of predictors relate to the interaction between the specific joke content and the specific receiver characteristics including whether the person is likely to understand the joke or consider it overly malicious.

One reason for imperfect model accuracies appears to be the difficulty of *measuring*, rather than predicting, individual instances of amusement reliably. People’s *average* reaction to humor stimuli can be measured with higher reliability but predicting these scores would disallow the usage of stimuli-level predictor variables that were of interest here (for measurement of humor appreciation, see^70^; for response aggregation, see^42^). Without reliable amusement responses, one cannot obtain flawless predictions for individual reactions. Situational fluctuations of amusement are well-known in the humor literature and integrating state constructs like a user’s current rating tendency pushed the models to (and sometimes beyond) the outcome’s temporal stability. To which degree the remaining errors are due to suboptimal model performance vs. poor measurement in the validation data remains uncertain and is methodologically challenging to address^71^.

The high predictive value of rating aggregates is encouraging for practitioners aiming to build humor applications and tools. For instance, recommendation engines that gradually collect user behaviors appear more promising than systems relying on tests of user personality. Further, many collaborative and content-based recommendation engines are designed to slowly capture optimal combinations of user and content characteristics. Based on the current research, integrating user tendencies with user-by-content interaction features is optimal to achieve a high recommendation performance. This is in line with findings achieved in a variance-decomposition approach^27^. Note that most humor appreciation theories acknowledge such interactivity in some way. For instance, the Benign Violation Theory forwards that *person-specific* perceptions of stimuli’s aggressiveness and benignity lie at the core of amusement^28^.

The funniness ratings used in the current work are similar to explicit content ratings requested by many online entertainment platforms. Importantly, many platforms also use implicit measures to assess user preferences (e.g., searches, view time). Measures of laughter or smiling, the closest behavioral companions to amusement, are usually not available to content providers. These behaviors are also rare and difficult to interpret in solitary settings^72^. Due to its social functions, laughter might come with an altered pattern of predictors (e.g., a higher predictive value of social personality traits) compared to the current study.

In order to obtain explicit preferences, entertainment platforms sometimes ask their consumers to describe what kind of content they would like to see. Our investigations show that this method works on occasion. Participant’s explicit attitudes (e.g., whether they like cheesy pick-up lines, or jokes about people’s appearance) were predictive of their later humor appreciation. However, these topic-specific questions did not perform consistently and would require effortful questionnaire work by users who want to be entertained. Similarly, correlations between personality/demographics and humor appreciation from the humor literature were largely replicated, but offer little incremental value in terms of prediction power. For instance, gender, age, and the Big 5, do show face-valid relationships (e.g., agreeable people disliking some aggressive jokes), however these relationships were generally weak and inconsequential for model performance. Thus, humor research on these variables continues to be insightful, but more so for theory development than practical applications.

We replicated the finding that people’s rating aggregates were more predictive than the stimuli’s rating aggregates^27^. However, the current study relativizes the previous authors’ advice to prioritize rater characteristics when predicting humor appreciation. Specifically, if we accepted the predictive value of prominent psychological constructs as relevant, one would be well advised to consider the success rate of the joke (among other raters) as well, because it outperformed all personality variables. Thus, we would extend the cited recommendation into “focus on audience tendencies, *specifically their past ratings, while not discarding average stimulus performance*”.

### Limitations and future directions

A general limitation that can be addressed gradually is the inclusion of additional predictor variables. Past research points towards certain interaction effects that were not included here but nonetheless steer humor appreciation: people’s dispositional preference for certain joke *structures*. An example is conservatism which steers people’s preference for a full resolution of incongruity at the end of jokes (as opposed to nonsense jokes^70^). Similarly, preferences for certain joke *formats* (e.g., text vs. video) can be tied to rater attributes (e.g., the ability to read) and can, depending on the stimuli and rater sample, become informative.

Additionally, we assume that additional *sources* of predictors, such as attributes of the joke teller or the relationship between joke teller and receiver can offer incremental gains. In past research, social cues and interpersonal perception affected the appreciation of the joke substantially (e.g., a halo effect of attractiveness^73^; gender dynamics between teller and listener^74^). To make this point clear, if you, the reader, feel like you could outperform the current models in predicting which of your contacts would laugh at your joke, you might rely on social cues that were not part of the current study. For instance, you are aware of the status dynamics at play when you speak to your personal contacts, and these affect humor responses significantly^75^. It is challenging to include such predictors in large-scale studies as the source of the joke has to be varied alongside the stimuli and rater characteristics. Here, we concentrated on performance humor, meaning jokes and images that can be implemented across social situations (e.g., content produced for online consumption). We did not include spontaneous humor that is afforded by specific social interactions, thus foregoing the predictive power of interpersonal predictor variables but also reducing sample size requirements, which were already substantial due to the highly-parametrized models we used. We assume that large web-scraping jobs, potentially including video recordings of social exchanges, alongside detailed annotations of the humor instances, could function to include such social and situational variables as predictors of humor appreciation.

We want to highlight the many analyses that can be conducted with the newly generated datasets. Close to 11.000 participants from multiple countries rated the humor stimuli. The dataset includes annotations of many demographic and psychological variables, allowing to re-test many statistical associations proposed in the literature. For some analyses, like the correlation between aggressiveness and funniness ratings, it might be reasonable to reformat and aggregate responses across either stimuli or subjects^42^. Data quality appears to be satisfactory (based on face-valid inter-item and inter-construct correlation, as well as successful attention checks, and sensitivity analyses), however it remains worthwhile to consider in which way measurement noise could have influenced our results. For instance, being able to measure predictors with higher reliability (e.g., moral internalization is assumed to be temporally unstable^49^) can increase their predictive power. People aiming to improve the performance of the prediction algorithms presented here, can extend the supplementary scripts through, for instance, advanced feature engineering.

## Conclusion

Humor appreciation is predictable. Variables like a person’s amusement propensity, joke popularity, and specific interactions between humor and rater characteristics allowed for prediction accuracies close to the suspected maximum. Main effects of broad psychological constructs were not useful to improve predictions. Humor research can be useful by forwarding the optimal range of predictors to content platforms. Field studies on such platforms would constitute a natural follow-up to the current work. Other research fields can apply a similar, prediction-focused approach to identify which of their studies and theories provide practical value.

### Supplementary Information

Supplementary Information.

## Data Availability

The datasets generated and analyzed during the current study are available in the OSF repository: https://shorturl.at/kmDGW.

## References

[1] Hofman JM, Watts DJ, Athey S, Garip F, Griffiths TL, Kleinberg J, Yarkoni T (2021). Integrating explanation and prediction in computational social science. Nature.

[2] Hofmann J, Platt T, Ruch W, Niewiadomski R, Urbain J (2015). The influence of a virtual companion on amusement when watching funny films. Motiv. Emot..

[3] Ruch W, Sander D, Scherer K (2009). Amusement. The Oxford Companion to the Affective Sciences.

[4] Heintz S (2020). Separating content and structure in humor appreciation. J. Individ. Differ..

[5] Ruch W, Rath S (1993). The nature of humor appreciation: Toward an integration of perception of stimulus properties and affective experience. Humor Int. J. Humor Res..

[6] Ruch W, Hehl F-J, Ruch W (2007). A two-mode model of humor appreciation: Its relation to aesthetic appreciation and simplicity-complexity of personality. The Sense of Humor: Explorations of a Personality Characteristic.

[7] Freud S (1905). Der Witz und Seine Beziehung zum Unbewußten (Jokes and its Relationship to the Unconscious).

[8] McCauley C, Woods K, Coolidge C, Kulick W (1983). More aggressive cartoons are funnier. J. Pers. Soc. Psychol..

[9] Gruner CR (2017). The Game of Humor: A Comprehensive Theory of Why We Laugh.

[10] Hobbes T (1994). Leviathan.

[11] Warren C, McGraw AP (2016). Differentiating what is humorous from what is not. J. Pers. Soc. Psychol..

[12] Wyer RS, Collins JE (1992). A theory of humor elicitation. Psychol. Rev..

[13] Ruch W, Köhler G, Van Thriel C (1996). Assessing the “humorous temperament”: Construction of the facet and standard trait forms of the state-trait-cheerfulness-inventory—STCI. Humor Int. J. Humor Res..

[14] Ruch W, Carrell A (1998). Trait cheerfulness and the sense of humour. Pers. Individ. Differ..

[15] Wicker FW, Thorelli IM, Barron WL, Willis AC (1981). Studies of mood and humor appreciation. Motiv. Emot..

[16] Ruch W, Köhler G, Van Thriel C (1997). To be in good or bad humour: Construction of the state form of the state-trait-cheerfulness-inventory—STCI. Pers. Individ. Differ..

[17] Galloway G, Chirico D (2008). Personality and humor appreciation: Evidence of an association between trait neuroticism and preferences for structural features of humor. Humor Int. J. Humor Res..

[18] Ku LC, Chang YT, Chen HC (2020). How do extraverts process jokes? An event-related potential study on humor processing. Brain Cogn..

[19] Köhler G, Ruch W (1996). Sources of variance in current sense of humor inventories: How much substance, how much method variance?. Humor Int. J. Humor Res..

[20] Moran JM, Rain M, Page-Gould E, Mar RA (2014). Do I amuse you? Asymmetric predictors for humor appreciation and humor production. J. Res. Pers..

[21] Ruch W, Deckers L (1993). Do extraverts, like to laugh?: An analysis of the situational humor response questionnaire (SHRQ). Eur. J. Pers..

[22] Deaner SL, McConatha JT (1993). The relation of humor to depression and personality. Psychol. Rep..

[23] Carretero-Dios H, Ruch W (2010). Humor appreciation and sensation seeking: Invariance of findings across culture and assessment instrument?. Humor.

[24] Forabosco G, Ruch W (1994). Sensation seeking, social attitudes and humor appreciation in Italy. Pers. Individ. Differ..

[25] Mendiburo-Seguel A, Páez D, Martínez-Sánchez F (2015). Humor styles and personality: A meta-analysis of the relation between humor styles and the Big Five personality traits. Scand. J. Psychol..

[26] Plessen CY, Franken FR, Ster C, Schmid RR, Wolfmayr C, Mayer AM, Tran US (2020). Humor styles and personality: A systematic review and meta-analysis on the relations between humor styles and the Big Five personality traits. Pers. Individ. Differ..

[27] Rosenbusch H, Evans AM, Zeelenberg M (2022). the relative importance of joke and audience characteristics in eliciting amusement. Psychol. Sci..

[28] McGraw AP, Warren C (2010). Benign violations: Making immoral behavior funny. Psychol. Sci..

[29] McGraw AP, Warren C, Williams LE, Leonard B (2012). Too close for comfort, or too far to care? Finding humor in distant tragedies and close mishaps. Psychol. Sci..

[30] Alvaro PK, Roberts RM, Harris JK (2013). A systematic review assessing bidirectionality between sleep disturbances, anxiety, and depression. Sleep.

[31] Fukuda, S., Matsuda, Y., Tani, Y., Arakawa, Y., & Yasumoto, K. Predicting depression and anxiety mood by wrist-worn sleep sensor. In *2020 IEEE International Conference on Pervasive Computing and Communications Workshops (PerCom Workshops)*, 1–6. (IEEE, 2020).

[32] Yarkoni T, Westfall J (2017). Choosing prediction over explanation in psychology: Lessons from machine learning. Perspect. Psychol. Sci..

[33] Rosenbusch H, Soldner F, Evans AM, Zeelenberg M (2021). Supervised machine learning methods in psychology: A practical introduction with annotated R code. Soc. Pers. Psychol. Compass.

[34] Bertero, D. & Fung, P. Deep learning of audio and language features for humor prediction. in *Proceedings of the Tenth International Conference on Language Resources and Evaluation (LREC'16)*, 496–501 (2016).

[35] Kamal, A. & Abulaish, M. Self-deprecating humor detection: A machine learning approach. in *International Conference of the Pacific Association for Computational Linguistics*, 483–494. (Springer, 2019).

[36] Mihalcea, R. & Strapparava, C. Making computers laugh: Investigations in automatic humor recognition. in *Proceedings of Human Language Technology Conference and Conference on Empirical Methods in Natural Language Processing*, 531–538 (2005).

[37] Yang, Z., Hooshmand, S. & Hirschberg, J. CHoRaL: Collecting humor reaction labels from millions of social media users. in *Proceedings of the 2021 Conference on Empirical Methods in Natural Language Processing*, 4429–4435 (2021).

[38] Annamoradnejad, I. & Zoghi, G. Colbert: Using bert sentence embedding for humor detection. arXiv:2004.12765 (2020).

[39] Shahaf, D., Horvitz, E. & Mankoff, R. Inside jokes: Identifying humorous cartoon captions. in *Proceedings of the 21th ACM SIGKDD International Conference on Knowledge Discovery and Data Mining*, 1065–1074 (2015).

[40] Hessel, J. *et al*. *Do Androids Laugh at Electric Sheep? Humor" Understanding" Benchmarks from The New Yorker Caption Contest*. arXiv:2209.06293 (2022).

[41] Winters T (2021). Computers learning humor is no joke. Harvard Data Sci. Rev..

[42] Ruch W (1995). Will the real relationship between facial expression and affective experience please stand up: The case of exhilaration. Cogn. Emot..

[43] Rammstedt B, John OP (2007). Measuring personality in one minute or less: A 10-item short version of the Big Five Inventory in English and German. J. Res. Pers..

[44] Lau C, Chiesi F, Saklofske DH (2022). The heart of humor: A network analysis of the temperamental basis of humor and humor personality traits. Pers. Individ. Differ..

[45] Lau C, Chiesi F, Hofmann J, Saklofske DH, Ruch W (2021). Development and linguistic cue analysis of the state-trait cheerfulness inventory: Short form. J. Pers. Assess..

[46] Chang C (2021). How morality judgments influence humor perceptions of prankvertising. Int. J. Advertis..

[47] Erzi S (2020). Dark Triad and schadenfreude: Mediating role of moral disengagement and relational aggression. Pers. Individ. Differ..

[48] Yam KC, Barnes CM, Leavitt K, Wei W, Lau J, Uhlmann EL (2019). Why so serious? A laboratory and field investigation of the link between morality and humor. J. Pers. Soc. Psychol..

[49] Aquino K, Reed A (2002). The self-importance of moral identity. J. Pers. Soc. Psychol..

[50] Hoyle RH, Stephenson MT, Palmgreen P, Lorch EP, Donohew RL (2002). Reliability and validity of a brief measure of sensation seeking. Pers. Individ. Differ..

[51] van Dongen JD, de Groot M, Rassin E, Hoyle RH, Franken IH (2022). Sensation seeking and its relationship with psychopathic traits, impulsivity and aggression: A validation of the Dutch Brief Sensation Seeking Scale (BSSS). Psychiatry Psychol. Law.

[52] Thorson JA, Powell FC (1991). Measurement of sense of humor. Psychol. Rep..

[53] Kerkkånen P, Kuiper NA, Martin RA (2004). Sense of humor, physical health, and well-being at work: A three-year longitudinal study of Finnish police officers. Humor Int. J. Humor Res..

[54] Carretero-Dios H, Delgado-Rico E, López-Benítez R, Acosta A (2023). Differential effects of affective arousal and valence on humor appreciation in female university students. Humor.

[55] Thompson ER (2007). Development and validation of an internationally reliable short-form of the positive and negative affect schedule (PANAS). J. Cross-cult. Psychol..

[56] Watson D, Clark LA, Tellegen A (1988). Development and validation of brief measures of positive and negative affect: The PANAS scales. J. Pers. Soc. Psychol..

[57] Burmeister JM, Carels RA (2015). Weight-related humor in the media: Appreciation, distaste, and anti-fat attitudes. Stigma Health.

[58] Martin RA, Puhlik-Doris P, Larsen G, Gray J, Weir K (2003). Individual differences in uses of humor and their relation to psychological well-being: Development of the Humor styles questionnaire. J. Res. Pers..

[59] Ruch W, Spielberger CD, Butcher JN (1992). Assessment of appreciation of humor: Studies with the 3 WD humor test. Advances in Personality Assessment, Vol. 9.

[60] Appinio. https://www.appinio.com/. Accessed 21 Jun 2023.

[61] Chen, T. & Guestrin, C. Xgboost: A scalable tree boosting system. In *Proceedings of the 22nd ACM SIGKDD International Conference on Knowledge Discovery and Data Mining*, 785–794 (2016).

[62] Borisov V, Leemann T, Seßler K, Haug J, Pawelczyk M, Kasneci G (2022). Deep neural networks and tabular data: A survey. IEEE Trans. Neural Netw. Learn. Syst..

[63] McElfresh, D. *et al*. *When Do Neural Nets Outperform Boosted Trees on Tabular Data?* arXiv:2305.02997. (2023).

[64] González S, García S, Del Ser J, Rokach L, Herrera F (2020). A practical tutorial on bagging and boosting based ensembles for machine learning: Algorithms, software tools, performance study, practical perspectives and opportunities. Inf. Fusion.

[65] Kuhn, M. & Wickham, H. *Tidymodels: A Collection of Packages for Modeling and Machine Learning Using Tidyverse Principles.* https://www.tidymodels.org. (2020).

[66] R Core Team. *R: A Language and Environment for Statistical Computing*. (R Foundation for Statistical Computing, 2022). https://www.R-project.org/.

[67] Guenter H, Schreurs B, Van Emmerik IH, Gijsbers W, Van Iterson A (2013). How adaptive and maladaptive humor influence well-being at work: A diary study. Humor.

[68] Ruch W (1997). State and trait cheerfulness and the induction of exhilaration. Eur. Psychol..

[69] Dunbar RIM, Launay J, Curry O (2016). The complexity of jokes is limited by cognitive constraints on mentalizing. Hum. Nat..

[70] Ruch W (2013). Assessment of appreciation of Humor: Studies with the 3 WD Humor test. Adv. Pers. Assess..

[71] Dejonckheere E, Demeyer F, Geusens B, Piot M, Tuerlinckx F, Verdonck S, Mestdagh M (2022). Assessing the reliability of single-item momentary affective measurements in experience sampling. Psychol. Assess..

[72] Provine RR (2004). Laughing, tickling, and the evolution of speech and self. Curr. Direct. Psychol. Sci..

[73] Cowan ML, Little AC (2013). The effects of relationship context and modality on ratings of funniness. Pers. Individ. Differ..

[74] McLachlan A (2022). The relationship between familiarity, gender, disagreement, and status and bouts of solitary and joint laughter. Curr. Psychol..

[75] Oveis C, Spectre A, Smith PK, Liu MY, Keltner D (2016). Laughter conveys status. J. Exp. Soc. Psychol..

