# Supplementary material for: Humor appreciation can be predicted with machine learning techniques
Source: Sci Rep. 2023 Nov 3;13:19035. doi: 10.1038/s41598-023-45935-1 (PMC10624684; doi:10.1038/s41598-023-45935-1)
Supplement: Supplementary file 1 — Supplementary Information. [file 41598_2023_45935_MOESM1_ESM.docx]

All materials are online under:

<https://osf.io/kvft2/?view_only=f889cb7f67614c3c9829f1e285821530>
